# Supplementary material for: Comparison of quality of life between elderly and non-elderly adult residents in Okuma town, Japan, in a post-disaster setting
Source: PLoS One. 2023 Feb 14;18(2):e0281678. doi: 10.1371/journal.pone.0281678 (PMC9928109; doi:10.1371/journal.pone.0281678)
Supplement: Attachment — Submitted filename: Response to Reviewers.docx [file pone.0281678.s002.docx]

We wish to express our thanks to the Editor and reviewers for providing feedback regarding our manuscript. We have revised the manuscript accordingly, and feel that these changes have improved the quality of the paper. Please find our point-by-point responses to all comments below.

Journal Requirements:

When submitting your revision, we need you to address these additional requirements.

1. Please ensure that your manuscript meets PLOS ONE's style requirements, including those for file naming. The PLOS ONE style templates can be found at
   <https://journals.plos.org/plosone/s/file?id=wjVg/PLOSOne_formatting_sample_main_body.pdf> and 
   <https://journals.plos.org/plosone/s/file?id=ba62/PLOSOne_formatting_sample_title_authors_affiliations.pdf>

We have reformatted our manuscript according to the PLOS ONE style requirements.

2. Please provide additional details regarding participant consent. In the ethics statement in the Methods and online submission information, please ensure that you have specified (1) whether consent was informed and (2) what type you obtained (for instance, written or verbal, and if verbal, how it was documented and witnessed). If your study included minors, state whether you obtained consent from parents or guardians. If the need for consent was waived by the ethics committee, please include this information. If you are reporting a retrospective study of medical records or archived samples, please ensure that you have discussed whether all data were fully anonymized before you accessed them and/or whether the IRB or ethics committee waived the requirement for informed consent. If patients provided informed written consent to have data from their medical records used in research, please include this information.

Informed consent was obtained using a cover letter, which explained the purpose of the study. Consent to participate was voluntary, and it was explained that participants who choose not to participate would face no disadvantages. Written informed consent was obtained from all participants through the return postage of the questionnaire. The results obtained was anonymous and individuals could not be identified. All study protocols were approved by the ethics committee of Nagasaki University Graduate School of Biomedical Sciences (approval No. 21082702-2, 25 November 2021). This study did not involve the participation of minors, the use of medical records, or any archived samples. We have added an ethics statement to the Methods section.

Revised manuscript (Materials and methods, L110-L115): The basis and purpose of the study were explained in a letter attached to the questionnaire, along with a privacy notice. Informed consent was obtained from all participants through the return postage of the questionnaire. All study protocols were approved by the ethics committee of Nagasaki University Graduate School of Biomedical Sciences (approval No. 21082702-2, 25 November 2021).

Review Comments and Author Response to Reviewer #1:

We would like to thank the reviewers for their valuable feedback, which has enabled us to improve the quality of our manuscript. Please find below our responses to the comments and revisions to the manuscript.

This manuscript purposed to examine the difference in the association among indicators of self-reported quality of life and other socio-psychologic factors, risk perception of radiation exposure including self-perceived knowledge level and desire to know about radiation, intention to return, and self-reported quality of life, comparing the association between the age groups of elderly and younger. The manuscript was written at an average level in the manner, but contains some crucial problems.

Thank you for your review. We have modified the manuscript accordingly, as follows.

1) The title of “The role of age in self-reported quality of life among elderly residents…” seems to imply that the investigated indicators were compared between age classes among the elderly and some specific analysis of the effects of age on those factors, but the contents of manuscript was simply the comparison between elderly and younger groups. So, the title may need to be modified to present the real contents.

Thank you for this comment. We agree that the title might be misleading and have revised it as follows: “Comparison of quality of life between elderly and non-elderly adult residents in Okuma town, Japan, in a post-disaster setting”.

2) It looked slightly ambiguous which association was focused among the investigated factors (aspects of investigations) in this study because there were many factors with various aspects. Authors may need to clarify their hypothesis about association rather than simple explore of association.

Thank you for this feedback. We apologize for the ambiguity regarding the scope of the paper and have made the following revisions, accordingly:

Revised manuscript (Introduction, L92-L100): This study aims to examine the role of age in self-reported quality of life among residents of Okuma town. Specifically, the study focuses on how the perception of risk of returning to reside in Okuma town and thus, the intention to return, differs between elderly and non-elderly residents. As demonstrated in previous studies, there is an association between risk perception, intention to return and quality of life among evacuees. We hypothesized that elderly residents would perceive lower risk than their younger counterparts, and thus express a higher intention to return. Additionally, we predicted a lower self-reported quality of life among elderly because of prolonged evacuation.

3) L59-62: The contents of this sentence is incorrect. The concept of radiation restriction for the public by ICRP was not solely by Mettler’s paper, but by substantially wide accumulation of knowledge. The level of radiation exposure for the public in the situation of “existent exposure” such as aftermath of nuclear accident is not “limit” but “reference level” that should adapt the real situation and should be changed in accordance with the efforts for improvement of environment. Authors should learn about those concept by the ICRP Publications 103 and 111. Otherwise this sentence should be deleted.

Thank you for this comment. We apologize for this erroneous sentence. We have studied ICRP publications 103 and 111, as suggested. We have made the following changes, accordingly:

Original manuscript (Introduction, L59-62): Based on evidence that an increase in cancer incidence and mortality occurs with exposure to radiation doses higher than 100 millisieverts (mSv) [5], the International Commission on Radiation Protection (ICRP) limits the range of annual exposure dose to the general population from 20 to 100 mSv during a nuclear disaster.

Revised manuscript (Introduction, L55-L67): Based on an aggregation of knowledge (including data from Hiroshima and Nagasaki atomic bomb survivors) indicating an increase in cancer incidence and mortality associated with exposure to radiation doses higher than 100 millisieverts (mSv), the International Commission on Radiological Protection (ICRP) proposed the use of bands and reference levels of radiation dose according to exposure scenarios as one of its radiological protection strategies [4, 5]. For the public under normal conditions, the ICRP recommends limiting annual exposure to a radiation dose below 1 mSv (the first band of 1 mSv or less). The second band (1-20 mSv) is applicable to the protection of persons residing in contaminated areas. Actions should be taken to limit individual annual effective residual dose towards the lower range of the band, with periodic reductions over time. Reference levels are adjusted based on relevant factors, such as contamination levels, decontamination progress, the sustainability of social, economic, and environmental life, and individual health status. The third band (20-100 mSv) is applicable to extreme cases, such as radiological emergencies.

4) L67: The word “declared” is inappropriate. Either body (WHO or UNCEAR) cannot and did not “declare” it. It might be replaced with “suggested” or some other.

Thank you for pointing this out. We apologize for the inappropriate wording and have revised the sentence as follows:

Revised manuscript (L71-L74): International governing bodies such as the World Health Organization (WHO) and the United Nations Scientific Committee on the Effects of Atomic Radiation (UNSCEAR) have published evidence suggesting no increases in the risks of cancer or related disease incidence, either presently or in the future, as a result of this disaster.

5) L97-100: The response rate to the survey was 21% (940/4400) and was too low. Reviewer thinks that it has been said in general that response rate of questionnaire surveys by municipality offices was around half in Japan. Recipients of the questionnaire might be reluctant with the too long list of questions, academic-oriented questions rather than cuddling up to their situation, or other reasons. Authors should have thought to keep the response rate as low response rate should induce serious selection bias.

Thank you for this comment. We acknowledge that the response rate is low and is a source of selection bias in the study. Please find our response below, which has been added to the Discussion section:

Revised manuscript (Discussion, L309-L322): Due to the nature of the 2011 disaster, various stakeholders, including government officials, academic institutions, and town councils have undertaken surveys of these residents. With time, residents naturally face survey fatigue, especially for voluntary questionnaires, compared with mandatory government-led surveys. A typical response rate for postal questionnaires, such as ours is around 20% [56]. The low response rate of 21% in the present study might have caused respondent bias if residents interested in returning or those who felt an attachment to their hometown were more likely to respond. Thus, the present results may represent the opinions of these residents specifically rather than all Okuma residents. As a population subset, elderly residents are more vulnerable and at higher risk of morbidity and mortality than other groups, especially during and after disasters. It is essential to support their rehabilitation and improve their quality of life after long-term evacuation. This research can assist Okuma Town and its residents in undertaking reconstruction efforts and in future disaster preparedness.

In addition, the response rate among each of elderly and younger groups needs to be reported.

We have added the response rate for both groups as follows:

Revised manuscript (Results, Demographic Characteristics, L158-L160): Of the 4,440 households, 940 responses were received, all of which were included in the analysis. Of the total residents who responded, 51.9% were elderly and 40.5% were non-elderly.

6) L158-166: It seems slightly curious that the authors asked the residents only about “desire to know more about treated water released form the FDNPP.” It seems necessary to compare it with the knowledge and attitudes of residents to radioactivity pollution on land and activities of countermeasures against it.

Thank you for this comment. We apologize for the ambiguity of the question. In April 2021, the Japanese government approved plans to discharge more than 1 million tons of treated water (which has been stored since the FDNPP accident) into the Pacific Ocean in 2023. We added this item to our questionnaire because we expected that residents might have a high level of anxiety about the perceived consequences of this decision, and that this issue might influence their intention to return to reside in Okuma town. Various related factors need to be considered for an in-depth analysis; therefore, this is an issue for consideration in future research.

Minor points:

1) L42-58: Explanation of the situation at the time of the accident seems to be too detailed although it seemed to be little associated with the contents of this manuscript. This might be shortened.

Thank you for your feedback. We have made the following changes accordingly:

Revised manuscript (Introduction L43-L54): As a result of the Great Japan Earthquake and tsunami on March 11, 2011, a nuclear accident occurred at the Fukushima Daiichi Nuclear Power Plant (FDNPP). Nuclear meltdowns occurred due to the loss of reactor core cooling, and hydrogen explosions took place in Units 1, 3, and 4 [1]. The disaster was characterized as a level 7 event (Major Accident) on the International Nuclear and Radiological Event Scale [2]. Consequently, radionuclides from the damaged plant spilled into the environment and an order to evacuate or remain indoors was issued to residents by the Prime Minister of Japan in his role as Director-General of the Nuclear Emergency Response Headquarters. At 20:50 that day, residents living within 2 km of the plant were ordered to evacuate. This order was extended to a 3 km radius, then to a 10 km radius, and finally, to a 20 km radius on March 12. These decisions resulted in a long-term and widespread evacuation of local residents. As Okuma housed units 1, 2, 3, and 4 of the FDNPP, all 11,505 residents were forced to evacuate.

2) Abstract, L32, 149, 160 (twice), 172, 175, 189, 246: The word “cohort” is inappropriate, but “group” is appropriate in this study design.

Thank you for this comment. We apologize for the incorrect usage of “cohort”, which we have replaced with “group”.

3) L217: “the odds” might be “odds ratio.”

Thank you for this comment. We have revised the manuscript, accordingly.

Review Comments and Author Response to Reviewer #2:

This study, which uses a questionnaire survey to understand the situation of residents in difficult-to-return areas affected by the nuclear disaster, is important not only for the development of post-disaster public health activities, but also for the efforts of local government administration.

The conclusion of the paper should briefly state the results of testing the research hypothesis based on the data obtained in the study and its interpretation. For this reason, the conclusions of this paper should be more compact. The content the authors wish to discuss in the conclusions section should be moved to the discussion section. It would be good to have a discussion on the impact of the measures on the significant differences found in terms of effect size.

Thank you for this feedback. We have shortened the Conclusions section and shifted the first paragraph to the Discussion.

Specific comment 1 Financial disclosure

The response could not be true because this research fund states that research proposals shall be evaluated by the Research and Survey Project Evaluation Committee and reflected in the review of research plans, etc.

This work was supported as a Research Project on the Health Effects of Radiation, organized by the Ministry for the Environment, Japan. The funders had no role in study design, data collection and analysis, decision to publish, or preparation of the manuscript. After consulting with the funders, we were informed that this description is sufficient.

Specific comment 2 Line 60

“Based on evidence that an increase in cancer incidence and mortality occurs with exposure 60 to radiation doses higher than 100 millisieverts (mSv) [5],”

Although this is a quoted and controversial point, there are studies where radiation risks have been found even for exposures with effective or equivalent doses of less than 100 mSv, and measures are being taken for radon based on epidemiological studies.

Ref.
Proposer D. J. Brenner, Opposer O. G. Raabe, Moderator J. C. McDonald. Is the linear-no-threshold hypothesis appropriate for use in radiation protection? Favouring the proposition. Radiat Prot Dosimetry. 2001;97(3):279-82; discussion 285.

Doss, M., Little, M. P., & Orton, C. G. (2014). Point/Counterpoint: low-dose radiation is beneficial, not harmful. Medical physics, 41(7), 070601.

Thank you for this comment. We agree that this point is a contentious one in the scientific community, and that studies have shown adverse effects from exposure to radiation doses below 100 mSv. To bring attention to low-dose effects of radiation, we have modified the text in the Introduction section to highlight the ICRP recommendations regarding banding and the reference levels for radiological protection stratified by dose range:

Revised manuscript (Introduction L55-L67): Based on an aggregation of knowledge (including data from Hiroshima and Nagasaki atomic bomb survivors) indicating an increase in cancer incidence and mortality associated with exposure to radiation doses higher than 100 millisieverts (mSv), the International Commission on Radiological Protection (ICRP) proposed the use of bands and reference levels of radiation dose according to exposure scenarios as one of its radiological protection strategies [4, 5]. For the public under normal conditions, the ICRP recommends limiting annual exposure to a radiation dose below 1 mSv (the first band of 1 mSv or less). The second band (1-20 mSv) is applicable to the protection of persons residing in contaminated areas. Actions should be taken to limit individual annual effective residual dose towards the lower range of the band, with periodic reductions over time. Reference levels are adjusted based on relevant factors, such as contamination levels, decontamination progress, the sustainability of social, economic, and environmental life, and individual health status. The third band (20-100 mSv) is applicable to extreme cases, such as radiological emergencies.

Specific comment 3 Line 98

“We distributed questionnaires to residents, who were able to receive mail from the municipal office (4,400 postal letters). The data collection period for this study was from 6 January to 2 March 2022. Of the 940 responses received, all were included in the analysis.”

The reviewer believes that a distinction should be made between methods and results. Shouldn't the actual numbers distributed and collected be presented as results, if you generalize the research to be conducted in the affected areas?

Thank you for your feedback. We have moved the following part of the text to the Results section:

Revised manuscript

(Methods, L108-L110): We distributed questionnaires to residents, who were able to receive mail from the municipal office. The data collection period was from 6 January to 2 March 2022.

(Results, Demographic Characteristics, L158-L159): Of the 4,440 households, 940 responses were received, all of which were included in the analysis

Specific comment 4 Line 99

Since this is a research study conducted with the cooperation of the town, the reviewer thought it would be a good idea to clearly state the protocol from an ethical standpoint regarding collaboration with the government and residents, as stated in the acknowledgments.

Thank you for this suggestion. In response, we have mentioned the details of the collaboration between the university and Okuma town hall in the Acknowledgements.

Revised manuscript (Acknowledgements, L346-L348): We would like to thank all study participants and staff members of the municipal government of Okuma. This study was conducted as a collaboration between Nagasaki University and Okuma Town Council.

Specific comment 5 Line 128

“Scores higher than 50 ± 10” Does the breadth of the criteria depend on some condition?

Thank you for this comment. The criteria for breadth were not dependent on any pre-determined condition. We have removed SD from the manuscript to avoid any confusion, as follows:

Revised manuscript (Methods, Questionnaire, L140): Scores higher than 50 were considered to indicate good health, based on mean values among the general population in Japan.

Specific comment 6 Line 143

To determine respondent bias, it would be helpful to present information on the demographic characteristics of not only those who responded to the survey, but also those who were subjects of this research.

Thank you for this suggestion. According to a survey conducted by the central government in 2021 on Okuma residents' intention to return, approximately 16% had already returned or wanted to return, 23% were undecided, and 57% had decided not to return, which is consistent with the results of this study. As of September 2019, the total population of Okuma was 10,300. This figure continues to decrease each year, compared to the total population of 11,500 prior to the disaster. In contrast, the population of residents aged 65 and over is 2,800 (elderly population rate, 27%), and the number in this group is expected to grow. We have revised the manuscript, accordingly (Discussion, L371).

Specific comment 7 Line 164

“There were no differences regarding the desire to learn the basics of radiation, but 76.8% of residents who expressed a desire to know more about treated water released from the FDNPP were elderly (p<.001).”

It would be better to make the wording more explicit such as “There were no differences between these age groups regarding the desire to learn the basics of radiation, but there are differences regarding the desire to know more about treated water released from the FDNPP with 76.8% of elderly residents expressed a desire to know with 71.5% of those under age 65 wanting to know (p<.001).”

Thank you for this comment. Based on your suggestion, we have modified the sentence as follows:

Revised manuscript (Results, L178-182): There were no differences between the two age groups regarding the desire to learn the basics of radiation, but there were differences regarding the desire to know more about treated water released from the FDNPP, where 76.8% of elderly residents and 71.5% of younger residents expressed a desire to know more (p<0.001).

Specific comment 8 Line 181

“There were no differences with regard to vitality.” Since p=0.063, it may be considered that the difference is not detected due to lack of power. If the authors claim that there is no difference, they need to show the result of equivalence testing.

Thank you for this suggestion. In response, we checked the boundaries for equivalence and found that the lower bound was –0.0016 and the upper bound was 0.133. We agree with the suggestion that a difference might not have been detected due to a lack of power, and have made the following changes, accordingly:

Original manuscript (Results, Quality of life, L181): There were no differences with regard to vitality.

Revised manuscript (Results, Quality of life, L197): Differences with regard to vitality could not be detected.

Specific comment 9 Line 186

Although understandable in the overall context, it would be better to clarify that the odds ratios are for each factor in the elderly compared to the under 65 age group. At least, Table 3 should be expressed in a way that it can be understood independently.

Thank you for this feedback. We apologize for the lack of clarity and have indicated the reference group in the revised manuscript as follows:

Revised manuscript (Results, Logistic regression, L202): Odds ratios regarding risk perception and quality of life were obtained for the elderly residents, with the younger residents as the reference group.

Table 3. Logistic regression – risk perception and quality of life for elderly residents

|  | **Variable** | **Reference** | **OR^1^** | **95% CI** |
| --- | --- | --- | --- | --- |
| **Risk perception** | **Sex** | Male/female | 1.125 | 0.848–1.492 |
|  | **Current economic status** | Uncomfortable/comfortable | 0.785 | 0.567–1.086 |
|  | **Anxiety regarding radiation health effects on future generations** | Yes/no | 1.368* | 1.024–1.827 |
|  | **Desire to know about FDNPP treated water** | Yes/no | 1.295* | 1.109–1.512 |
| **Quality of life** | **Sex** | Male/female | 1.297 | 0.971–1.734 |
|  | **Current economic status** | Uncomfortable/comfortable | 0.893 | 0.745–1.071 |
|  | **Physical component score** | <50/≥50 | 2.229* | 1.642–3.025 |
|  | **Mental component score** | <50/≥50 | 1.163 | 0.859–1.574 |
|  | **Interaction with friends** | Yes/No | 1.337* | 1.165–1.534 |

OR: odds ratio; CI: confidence interval; *p-value <0.05

^1^: Reference group is young residents (age <65 years)

Specific comment 10 Line 209

Although, as the authors state, no heritable effects have been clearly found in humans, heritable effects of radiation have been found in mammals. Research on heritable epigenetic effects is also needed in humans.

As another point of contention, is ANXIETY itself a problem? Anxiety should be one of the most important emotions in humans.

Niwa, O. Induced genomic instability in irradiated germ cells and in the offspring; reconciling discrepancies among the human and animal studies. Oncogene 22, 7078–7086 (2003) doi:10.1038/sj.onc.1207037

European Commission, Directorate-General for Energy, Epigenetic effects : potential impact on radiation protection : proceedings of a scientific seminar held in Luxembourg on 8 November 2017, Publications Office, 2019, <https://data.europa.eu/doi/10.2833/731758>

Thank you for this comment. We agree with the reviewer that heritable effects have been demonstrated in animal studies, and there is a need for more large-scale and robust research among human subjects. We have added the following comments regarding anxiety in the Discussion section accordingly:

Revised manuscript (Discussion, L232 – L239): Anxiety is a meaningful emotion experienced by humans as it enables individuals to perceive risk and avoid hazardous situations. Feelings of anxiety and risk perception are also substantially dependent on an individual's past experiences and the pattern of that individual’s mental heuristics [36]. As such, general feelings of anxiety are normal and temporary in most individuals. However, after experiencing a disaster, a long-term evacuation and the possible resulting PTSD, such feelings of anxiety might be uncontrollable and persistent, leading to misjudgment of risk and long-term detrimental effects on health and quality of life.

Specific comment 11 Line 209

“It is essential to reiterate and underscore these findings in a clear and concise manner to the elderly to prevent misunderstandings and inflated risk perceptions.”
Is it a risk communication issue that the facts are not being communicated in a way that is easily understood? Rather, isn't the background issue more important?

Thank you for this comment. The social and cultural background of Japan cannot be ignored when considering residents’ perception of radiation. Because of Japan’s history with the Hiroshima and Nagasaki atomic bombings, there has been a cultural fear of radiation that dates back several generations. As a result of the FDNPP disaster, and the subsequent mishandling of the evacuation order by the authorities, this fear was heightened. Through continual educational interventions in schools and promotion of a practical radiological culture *(Wada Y, et al. Developing 'practical radiological culture'. A proposal of 'Kizuna square' in Fukushima. Nippon Genshiryoku Gakkai-Shi. 2013;55(9):520-3)*, initiatives have been underway to improve residents’ radiation literacy and enable them to live their daily lives in Fukushima without fear. However, various studies *(Matsunaga H, et al. Intention to return to the town of Tomioka in residents 7 years after the accident at Fukushima Daiichi Nuclear Power Station: a cross-sectional study. Journal of Radiation Research. 2019;60(1):51-8.)* have demonstrated that younger residents, especially young mothers, have a higher risk perception of the genetic effects of radiation and are also less likely to consult with radiation experts, compared with older males. Thus, the sociocultural aspect of risk perception at the sub-population level requires further study and targeted interventions.

Specific comment 12 Line 222

While it is true that the hydrogen explosions damaged the reactor buildings, a large amount of radioactive material was released into the environment prior to the hydrogen explosion, with the largest release coming from Unit 2, which did not experience a hydrogen explosion.

Thank you for pointing this out. We have revised the following sentence:

Revised manuscript (Discussion, L251): Groundwater and rainwater entered broken buildings housing the reactors that were damaged by the hydrogen explosion, resulting in the production of water imbued with high concentrations of radioactive materials.

We have also added the following information to the Introduction section:

Revised manuscript (Introduction, L47): Consequently, radionuclides from the damaged plant spilled into the environment and an order to evacuate or remain indoors was issued to residents by the Prime Minister of Japan in his role as Director-General of the Nuclear Emergency Response Headquarters.

Specific comment 13 Line 225

“This was achieved for all radionuclides, with the exception of tritium.” I-129 was found to be above the standard concentration in April 2019. Several exceedances of standard concentrations were also confirmed for Tc-99.

Thank you for this feedback. For the sake of clarity, we have specified the time period and referenced a leaflet from the Ministry of Economy, Trade and Industry which states that “…most of the radioactive materials are removed by ALPS to meet the regulatory standards with an exception of tritium”.

Reference: <https://www.meti.go.jp/english/earthquake/nuclear/decommissioning/atw.html>

Original manuscript (Discussion, L225): This was achieved for all radionuclides, with the exception of tritium.

Revised manuscript (Discussion, L256): As of 2022, the Ministry of Economy, Trade, and Industry, Japan and the IAEA stated that this was achieved for almost all radionuclides with the exception of tritium.

Specific comment 14 Line 280

“Okuma; however, the sample sizes in the former group are limited…”Respondent bias is more important in interpreting the results than the small response rate, which is subject to chance variation.

Thank you for pointing this out. We agree that respondent bias is a concern. In response, we have made the following revisions in the Discussion section that address this limitation:

Revised manuscript (Discussion, L313-L321): The low response rate of 21% in the present study might have caused respondent bias if residents interested in returning or those who felt an attachment to their hometown were more likely to respond. Thus, the present results may represent the opinions of these residents specifically rather than all Okuma residents. As a population subset, elderly residents are more vulnerable and at higher risk of morbidity and mortality than other groups, especially during and after disasters. It is essential to support their rehabilitation and improve their quality of life after long-term evacuation. This research can assist Okuma Town and its residents in undertaking reconstruction efforts and in future disaster preparedness.

Specific comment 15 Line 281

“Future studies should also focus on exploring residents' fears regarding disposal of the treated water from the FDNPP.. ”Are residents really feared? And, if so, is it the root problem of this issue that residents are fearing?

Thank you for your feedback. We apologize for the ambiguity and have changed the word “fear” to “concerns”. From speaking informally with the residents, we have come to realize that they are concerned regarding the effects of the disposed water on marine life in the oceans. Also, residents who are employed in the fishery industry have concerns that seafood will not be purchased from these areas due to the fear of tritium contamination. A few residents were under the misconception that tritium will contaminate the town’s drinking water sources. Thus, residents perceive a certain degree of risk regarding living in Okuma, which borders the ocean.

Original manuscript (Discussion, L281): Future studies should also focus on exploring residents' fears regarding disposal of the treated water from the FDNPP, as this will also affect residents’ intentions to return.

Revised manuscript (Discussion, L328): Future studies should also examine residents' concerns regarding the disposal of treated water from the FDNPP, as this may affect intentions to return.
