# Supplementary material for: Comparison of quality of life between elderly and non-elderly adult residents in Okuma town, Japan, in a post-disaster setting
Source: PLoS One. 2023 Feb 14;18(2):e0281678. doi: 10.1371/journal.pone.0281678 (PMC9928109; doi:10.1371/journal.pone.0281678)
Supplement: Attachment — Submitted filename: Response to Reviewers_2.docx [file pone.0281678.s003.docx]

Author Response to Editorial Comments and Reviewers:

We thank both reviewers for their time and effort in providing feedback. We have addressed all the points raised, to the best of our abilities. Please find our point-by-point responses (in purple) and manuscript revisions (in blue) below.

Reviewer #1: The manuscript has been significantly improved. But the low response rate to the survey is still the crucial problem of this report. Even if the response rate of around 20% is inevitably as authors said (L312-316), small differential distribution of background factors between elderly and younger groups could eventually turn the results upside down by confounding and/or biases. It is essential that the difference in response rate and characteristics of responders between the two groups should be examined, and it would help understanding the results by those who know the characteristics of the subject population well, for examples, members of the local community. But, the reviewer is still afraid if international readers might be misled or confused.

Thank you for these comments. We agree that differences between the elderly and younger residents and between responders and non-responders are important information that can give better context to the study results. Accordingly, for further clarification and to avoid confusion, we have provided these details in our manuscript. In addition, we would like to include the following points that we feel are relevant regarding the current situation of Fukushima evacuees.

Towns that lifted their evacuation orders early (2012), such as Kawauchi village, witnessed a return of almost 80% of their residents, but the same was not true for towns closer to the power plants where evacuation orders remained. Okuma was one of the towns in which the damaged nuclear reactors were located, and the evacuation order remained until 2019. Even in 2022, the evacuation order has not been fully lifted.

During the eight years of evacuation faced by Okuma residents, many families, especially those with young children and working professionals, settled in their evacuated areas and integrated themselves into new communities at their new places of residence. A survey conducted by the Okuma town council found that an interest in returning was expressed by mostly elderly evacuees. Our previous surveys conducted in Tomioka (Matsunaga H, et al. Journal of Radiation Research. 2019.) and Kawauchi (Matsunaga H, et al. International Journal of Environmental Research and Public Health. 2022.) found that older men who were not living with children were most likely to be interested in returning, and this group had a higher interest in seeking information regarding living in their hometowns, including participating in communication sessions related to risk communication and town recovery efforts.

Although the response rate to the questionnaire has decreased over time (as demonstrated in the Fukushima Health Management Survey responses, from 40% in 2012 to 20% most recently), our aim in conducting this research was to aid in the rehabilitation of Okuma town, including respecting the wishes of those residents who do not wish to return. A small proportion of elderly residents reported a wish to return, but the majority did not. This is perhaps a message in and of itself, indicating the need to refocus efforts to attract new residents into the town. Nevertheless, the desires of elderly residents who wish to return cannot be disregarded. This information is valuable to the Okuma town council for facilitating the return of residents, especially older people who wish to do so, while also providing insights to the international community regarding the realities of post-nuclear accident rehabilitation.

The following points need to be considered to revise.

1) L69-71: Figures of this sentence look like that the number of subjects is those fallen in the band while the percentage is for the subjects from zero dose to the upper limit. eg 1284 seems to be the subjects with 1 to <2 mSv, but 96.8% seems to be for zero to <2 mSv.

Thank you for pointing out this error, which we have corrected as follows.

Introduction, L68-72: “In July 2011, 4 months after the accident, the prefectural government measured radiation exposure doses in 4814 Okuma residents and found that 3374 (70.1%) received <1 mSv, 1284 (26.7%) received 1–2 mSv, 112 (2.3%) received 2–3 mSv, and 99.6% of residents were exposed to doses of 5 mSv or less [6]”

2) L97-100: The release of treated water to the sea needs to be included in the hypothesis because the question was specific to this survey.

We have included the subject of treated water into the hypothesis of the paper as follows.

Introduction, L101-102: “We predict that the concern regarding the release of treated water will be equally high among all Okuma residents.”

3) L157-159. (Important) Authors seemed to misunderstand the “response rate.” Response rate is “number of respondents / number of target subjects” for each of elderly and younger groups. Authors showed the proportion of elderly and younger groups only among the responders. The number of target subjects might be shown at the top row of Table 1. In addition, as separate for each sex needs to be indicated as the number of target subjects by sex should be known.

We thank the reviewer for this comment. Surveys were sent to all households that were able to receive mail from the municipal office, and one (or two) family member above the age of 20 was requested to fill out the survey. Our surveys were sent to almost every household listed with the town. From the statistical data maintained by the town council, we only have information regarding the number of residents and not the number of members per household. Thus, we are unable to provide an exact response rate. Therefore, we have added the following information on response rates, based on demographic data maintained by the town council, to the Methods and Results sections, to show differences between elderly and non-elderly and between respondents and non-respondents as accurately as possible.

Methods, L110-115: “We distributed questionnaires to residents able to receive mail from the municipal office. The total population in Okuma was approximately 10,100 (5,000 males and 5,100 females), and the number of total households was approximately 4,800. The distribution of the elderly and non-elderly aged 20 and over in national statistics of Okuma town was 34.7% (32.0% elderly males and 37.2% elderly females) and 46.5% (48.0% non-elderly males and 45.0% non-elderly females) respectively.”

Results, L163-165: “From the 4,440 households, 940 responses were received. Of the total residents who responded, 51.9% were elderly and 40.5% were non-elderly. After excluding 71 responses in which age was missing, 869 were included in the analysis.”

4) Table 1. The sum of 4 cells of sexes and age groups was 869 (248+240+187+194), which is different from 940 in L157 although all of them were included in the analysis.

Thank you for pointing this out. We have revised the cells in Table 1.

5) L216-266: This part looks like one paragraph. It is too long and looks including quite different aspects, so had better be divided into 4 paragraphs: L216-225, L225-241, L241-249, L249-266.

We appreciate this suggestion and have made these changes in the manuscript.

6) L318: “other groups” looks ambiguous. Is it “younger group”? Why the word is plural form?

We have changed this to “younger group” to avoid confusion.

7) L307-329: This paragraph might be divided at L316, or more.

We appreciate this comment and have split the paragraph as suggested.

8) L323: “the central government” should be “the central government of Japan.”

We have made this change as suggested.

Discussion, L332-336: “According to a survey conducted by the central government of Japan in 2021 on Okuma residents' intention to return, approximately 16% had already returned or wanted to return, 23% were undecided, and 57% had decided not to return, which is consistent with the results of this study.”

9) L322-325: This sentence needs a citation (reference literature).

We have cited a relevant study as requested.

10) L327: Limited sample size would give rise to some selection bias in responders, but it would not give rise to biases promptly in the results of comparison when background of the compared groups was not differential. we don't have the background information of non-responders.

Thank you for this comment. Among the responders, the significant differences between elderly and non-elderly responders were that most elderly lived in 2-person families and most younger residents were living in families consisting of 3 or more persons. Most elderly were not employed (due to their age) and most younger residents were employed. These differences form the basis of our discussion, where a larger number of elderly residents who were not working or living with more than one other person were more likely to seek information regarding living in Okuma town.

Regarding the non-responders, the background information we could obtain was related to age and sex distribution in the dataset of Okuma town council as of December 2021. The distribution of elderly and younger adult residents was 34% and 46% respectively, and our results showed a distribution of 52% and 41%. There was a higher response rate from the elderly. The distribution of male to female residents was 49% and 51% in Okuma town, and 48% and 52% in our study.

11) L361, L364: Publication numbers (111 and 103 for each) would be helpful for readers.

We have added the publication numbers in the manuscript, as suggested.

12) Reference: Style/format need to be checked thoroughly.

We have reformatted all references according to the *PLOS ONE* guidelines.

Reviewer #2: Specific comment 1 Financial disclosure

The response could not be true because this research fund states that research proposals shall be evaluated by the Research and Survey Project Evaluation Committee and reflected in the review of research plans, etc.

Additional comments

Since the evaluation committee shall evaluate the study design, and the evaluation shall be reflected in the revision of the study design, if the evaluation committee had made any contribution to this study, it should be clearly stated.

<https://www.env.go.jp/chemi/rhm/study/R3_01.html>

Thank you for this comment. We have revised the text as follows: “This work was supported as a Research Project on the Health Effects of Radiation, organized by the Ministry for the Environment, Japan. An Evaluation Committee was established for this project, which implemented appropriate research management by reviewing progress and implementation and providing advice when necessary. The progress and implementation for this study has also been checked. The funder was not involved in study design, data collection and analysis, or preparation of the manuscript.”

Specific comment 2 Line 60 of the original manuscript

“Based on evidence that an increase in cancer incidence and mortality occurs with exposure to radiation doses higher than 100 millisieverts (mSv) [5],”

Although this is a quoted and controversial point, there are studies where radiation risks have been found even for exposures with effective or equivalent doses of less than 100 mSv, and measures are being taken for radon based on epidemiological studies.

Excerpts from the authors' responses

For the public under normal conditions, the ICRP recommends limiting annual exposure to a radiation dose below 1 mSv (the first band of 1 mSv or less). areas. Actions should be taken to limit individual annual effective residual dose towards the lower range of the band, with periodic reductions over time.

Additional comments

As Reviewer 1 stated, reference levels rather than dose limits are applied in the existing exposure situation, so it is better to use different terminology.

We appreciate this suggestion. We have changed the terminology from “dose limits” to “reference levels”.
