# Supplementary material for: Comparison of quality of life between elderly and non-elderly adult residents in Okuma town, Japan, in a post-disaster setting
Source: PLoS One. 2023 Feb 14;18(2):e0281678. doi: 10.1371/journal.pone.0281678 (PMC9928109; doi:10.1371/journal.pone.0281678)
Supplement: Attachment — Submitted filename: response to reviewers_3.docx [file pone.0281678.s004.docx]

Author Response to Editorial Comments and Reviewer Comments:

We thank the reviewers for their time and effort in providing feedback. We have addressed all the points raised to the best of our ability. Please find our point-by-point responses (in purple) and manuscript revisions (in blue) below.

Reviewer #1:

The manuscript is continuously improved. But, the followings are still crucial problems.
1) The 1st point that authors replied [Towns that lifted their evacuation orders early (2012), the realities of post-nuclear accident rehabilitation] needs to be explained not only to reviewer but to readers. This reviewer could not think that the contents are incorporated sufficiently in the manuscript.

Thank you for the comment. We have included this information in the Introduction and Discussion sections of our manuscript for the benefit of readers (L75-86, L102-108, L394-404).

2) Methods of mailing of questionnaires are still ambiguous. It does not matter whether the questionnaires are sent individually or per household, as long as the questionnaires are distributed for all household members aged 20 years or older (adults). If the questionnaires sent to a household are insufficient for the number of people in that household, that is a big problem. A clear statement on this point is needed on the manuscript. Not only in reply to reviewers.

We appreciate your suggestions on explain the methodology of mailing questionnaires more clearly. The town council has information regarding the total number of residents, but not about the number of members per household. In addition, we do not have information on the names and postal addresses of individual residents. However, in order to cover our target sample of residents aged 20 and older, we distributed the questionnaires through the municipal office to most households in the study area with two sets per household. In the instructions provided in the questionnaire, we asked that if the household consisted of one target participant that the other questionnaire be discarded. If there were three or more study participants in the household, additional questionnaires would be mailed to them. Thus far, we found that there was no confusion or complaints from residents regarding this distribution method, but we will proactively make changes based on residents’ feedback. In response to the reviewer’s comment, we revised the manuscript as follows:

Materials and methods, Participants, L127-133:

“The study participants were current residents and evacuees aged ≥20 years who held resident cards for Okuma town as of December 2021 and who were able to receive mail from the municipal office. Questionnaires were distributed by the municipal office to most households, with two sets per household. In the instructions provided in the questionnaire, we asked that if the household consisted of one study participant, that the other questionnaire be discarded. If there were three or more target participants in the household, additional questionnaires would be mailed to them”

3) Authors need to show the number of residents with age of 20 to 64 years and 65 years or older by sex (approximate numbers would be accepted). Then the (rough) response rates need to be shown specifically for the numbers of respondents by sex and age class (248, 187, 240, and 194 in Table 1).

We thank the reviewer for this comment. We have made this correction in the manuscript as follows:

Materials and methods, Participants, L137-141:

“The total population in Okuma was approximately 10,100 (5,000 males and 5,100 females), and the number of total households was approximately 4,800. Excluding residents aged less than 19 years, the distribution of the elderly and non-elderly, according to national statistics for Okuma town was 3500 persons or 34.7% (1,600 or 32.0% elderly males and 1,900 or 37.2% elderly females) and 4,700 persons or 46.5% (2,400 or 48.0% non-elderly males and 2,300 or 45.0% non-elderly females), respectively.”

Results, Demographic Characteristics, L195-199:

“After excluding the 71 responses for which age was missing, 869 were included in the analysis. The response rates for elderly were 15.5% (248 / 1,600) for males and 12.6% (240 / 1,900) for females, and for non-elderly were 7.8% (187 / 2,400) for males and 8.4% (194 / 2,300) for females. Among all respondents, 51.9% were elderly and 40.5% were non-elderly”

4) L102: “among all Okuma residents” is ambiguous. Need to say “between sexes, ages, or other characteristics/attributes.”

We thank the reviewer for this comment. We have made this correction in the manuscript as follows:

Introduction, L116-120:

“We predict that the concern regarding the release of treated water will be equally high among all Okuma residents, regardless of sex, age or any other demographic characteristics or attributes”

Minor point: the first letter of “publications 103, 111” needs to be capitalized.

We have made the suggested correction (Introduction, L60).
